# Propagation dynamics of spin excitations along skyrmion strings

**DOI:** 10.1038/s41467-019-14095-0

**Published:** 2020-01-14

**Authors:** S. Seki, M. Garst, J. Waizner, R. Takagi, N. D. Khanh, Y. Okamura, K. Kondou, F. Kagawa, Y. Otani, Y. Tokura

**Affiliations:** 1 Department of Applied Physics, University of Tokyo, Tokyo, 113-8656 Japan; 2 Institute of Engineering Innovation, University of Tokyo, Tokyo, 113-8656 Japan; 3 RIKEN Center for Emergent Matter Science (CEMS), Wako, 351-0198 Japan; 4 PRESTO, Japan Science and Technology Agency (JST), Kawaguchi, 332-0012 Japan; 5 Institut für Theoretische Physik, Technische Universität Dresden, 01062 Dresden, Germany; 6 Institut für Theoretische Festkörperphysik, Karlsruher Institut für Technologie, 76131 Karlsruhe, Germany; 7 Institut für Theoretische Physik, Universität zu Köln, Zülpicher Str. 77a, 50937 Köln, Germany; 8 Institute for Solid State Physics, University of Tokyo, Kashiwa, 277-8581 Japan

**Keywords:** Magnetic properties and materials, Spintronics

## Abstract

Magnetic skyrmions, topological solitons characterized by a two-dimensional swirling spin texture, have recently attracted attention as stable particle-like objects. In a three-dimensional system, a skyrmion can extend in the third dimension forming a robust and flexible string structure, whose unique topology and symmetry are anticipated to host nontrivial functional responses. Here we experimentally demonstrate the coherent propagation of spin excitations along skyrmion strings for the chiral-lattice magnet Cu_2_OSeO_3_. We find that this propagation is directionally non-reciprocal and the degree of non-reciprocity, as well as group velocity and decay length, are strongly dependent on the character of the excitation modes. These spin excitations can propagate over a distance exceeding 50 μm, demonstrating the excellent long-range ordered nature of the skyrmion-string structure. Our combined experimental and theoretical analyses offer a comprehensive account of the propagation dynamics of skyrmion-string excitations and suggest the possibility of unidirectional information transfer along such topologically protected strings.

## Abstract (web summary)

The propagation dynamics of skyrmions in three dimensions have attracted increasing attention. Here, the authors demonstrate the coherent and non-reciprocal propagation of spin excitations along skyrmion strings for the chiral-lattice magnet Cu_2_OSeO_3_ over a distance exceeding 50 μm.

## Introduction

**Fig. 1:**
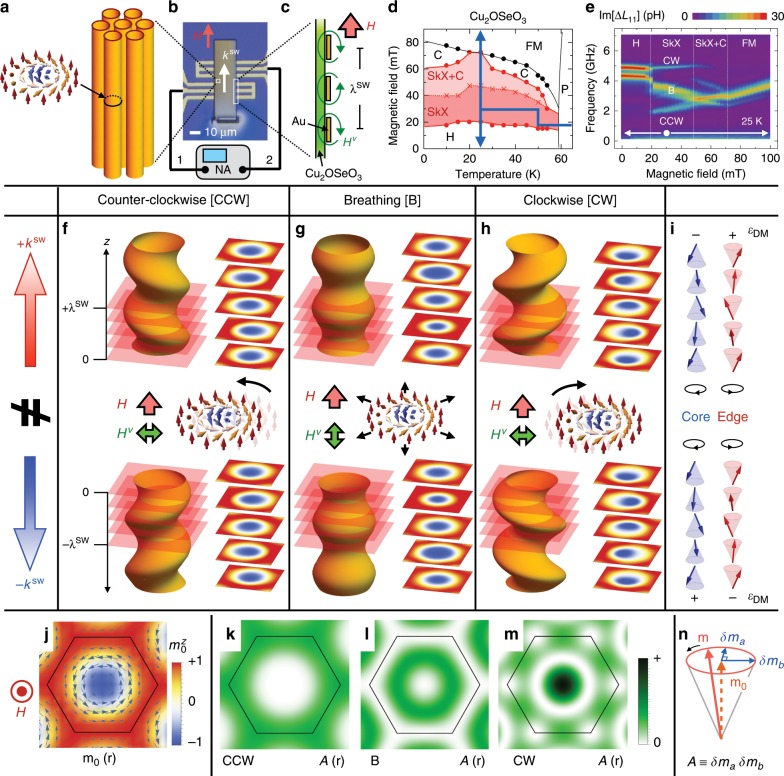
Propagating excitation modes on skyrmion strings. **a** Schematic illustration of skyrmion strings. **b**, **c** Top view optical image of device structure and side view illustration of a coplanar waveguide, where the AC current injected from network analyzer (NA) generates oscillating magnetic field *H*^*ν*^ and causes spin excitation in the neighboring Cu_2_OSeO_3_. **d** *H*–*T* magnetic phase diagram for Cu_2_OSeO_3_, obtained with the cooling path shown by the arrows. SkX, C, H, FM, and P represent the skyrmion lattice, conical, helical, ferromagnetic, and paramagnetic states, respectively. **e** The corresponding *H*-dependence of magnetic resonance spectra *ΔL*_11_ at 25 K. **f**–**h** Schematic illustration of CCW, breathing, and CW excitation modes on skyrmion strings. The central part represents the local oscillation manner of skyrmion at the *z* = 0 plane. The upper and lower parts are the snapshot images describing how the spin excitation launched at *z* = 0 propagates on the skyrmion strings, along the  ±*z* direction parallel and antiparallel to *H*, respectively. The cross-sectional images describing the size and position of skyrmion at selected *z*-planes (shown by red layers) are also indicated. **i** The direction of local magnetic moment at the core and edge position of skyrmion in each *z* layer. Black rounded arrows denote the sense of local moment precession in the time domain, and ± symbols indicate the sign of local DM energy gain *ϵ*_DM_. **j**–**m** Calculated spatial distribution of local magnetization direction **m**_0_(**r**) in the ground state and local amplitude of elliptical magnetization precession ${\mathcal{A}}({\bf{r}})$ for the CCW, B, and CW excitation modes in the SkX phase. In **j**, the arrows and background color (${m}_{0}^{z}({\bf{r}})$) represent the in-plane and out-of-plane component of **m**_0_(**r**), respectively. **n** Schematic illustration of local magnetization dynamics, described by ${\bf{m}}={{\bf{m}}}_{0}+{\rm{Re}}[(\delta {m}_{\mathrm{{a}}}{{\bf{e}}}_{\mathrm{{a}}}+i\delta {m}_{\mathrm{{b}}}{{\bf{e}}}_{\mathrm{{b}}})\exp [-i2\pi \nu t]]$ with **e**_a_ and **e**_b_ being the orthogonal unit vectors normal to **m**_0_. Here we define the local elliptical precession amplitude ${\mathcal{A}}\equiv \delta {m}_{\mathrm{{a}}}\delta {m}_{\mathrm{{b}}}$, whose spatial distribution for each mode is plotted in **k**–**m**.

Recently, the concept of a magnetic skyrmion, i.e., topologically stable spin configuration whose spins point in all of the directions wrapping a sphere, has attracted enormous attention^1–4^. In a magnetically two-dimensional system, a skyrmion appears as a vortex-like swirling spin texture with particle nature as shown in Fig. 1j. The stable particle nature of the skyrmion suggests its potential application as a magnetic bit for future data storage devices, and the nontrivial topology and symmetry of the skyrmion also cause exceptional electromagnetic responses^3,4^.

In three-dimensional systems, the skyrmion can form a string structure by extending in the third dimension, which consists of the uniform stacking of two-dimensional skyrmions along the string direction (Fig. 1a)^5,6^. Skyrmion strings can be considered as an analog of the vortex line in superfluids^7,8^, type-II superconductors^9^, and trapped dilute-gas Bose–Einstein condensates^10^ or the cosmic string in the universe^11^. They are all flexible and some of these strings are proposed to host a resonant oscillation mode propagating through the string path. This implies the possible coherent signal transfer along skyrmion strings, whereas the propagation character of their excitations has rarely been investigated before.

Experimentally, such skyrmion strings appear in a series of bulk magnets with chiral cubic atomic lattice. The examples are metallic B20^1,2^ or *β*-Mn-type Co-Zn-Mn^12^ alloys and insulating Cu_2_OSeO_3_^13,14^, the latter of which is the target of this work. In these materials, the Dzyaloshinskii–Moriya (DM) interaction is the key for the skyrmion formation. For a limited temperature (*T*) range, these compounds host a hexagonal lattice of skyrmion strings aligned along the static magnetic field (*H*) direction (Fig. 1a). This skyrmion crystal (SkX) phase is predicted to host three distinctive magnetic resonance modes^15–17^ (central panels in Fig. 1f–h), i.e., the counter-clockwise (CCW) and clockwise (CW) rotational modes both excited by an oscillating magnetic field *H*^*ν*^ ⊥ *H*, as well as the breathing (B) mode excited by *H*^*ν*^∥*H*, which have recently been identified by magnetic resonance experiments^16,18^. However, these previous works mostly focused on the character of non-propagating uniform excitations with wave number *k*^SW^ = 0. To understand their propagation character, the employment of a different experimental approach sensitive to the *k*^SW^ ≠ 0 regime, as well as the theoretical identification of their dispersion relation, is essential.

In this work, we investigated the propagation characteristics of such spin excitations along skyrmion strings. We find that the counter-propagating spin excitations show different propagation behavior and the degree of non-reciprocity, as well as the associated group velocity and decay length, are strongly dependent on the character of the excitation modes. These experimental features are well reproduced by our theoretically calculated dispersion relations. The observed decay lengths exceed 50 μm, reflecting the excellent long-range order of the skyrmion-string structure. The present results revealed the peculiar propagation dynamics of skyrmion-string excitations and suggest that skyrmion string can be a good medium for magnon transport with unique functionalities.

## Results

### Propagation dynamics of spin excitations

The detailed operating principle of the present measurement technique (propagating spin-wave spectroscopy) is provided in refs. ^19,20^. Figure 1b, c indicate the device structure employed in this study, where a plate-shaped Cu_2_OSeO_3_ single crystal with thickness *b* ≈  2 μm is placed on top of a pair of Au coplanar waveguides (CPWs) fabricated on a Si substrate. When the oscillating electric current of frequency *ν* is injected into a CPW, the generated oscillating magnetic field *H*^*ν*^ induces resonant spin excitations in the neighboring Cu_2_OSeO_3_ sample as shown in Fig. 1c. These spin excitations propagate along the sample and induce an oscillating electric voltage in the CPWs through the inverse process. By measuring the magnetic contribution to the self-inductance *ΔL*_11_ and mutual inductance *ΔL*_nm_ spectra (with *m* and *n* representing the port number used for the excitation and detection, respectively) for CPWs using a vector network analyzer (NA), we can directly evaluate the local excitation character and propagation character of spin excitations, respectively. Unless specified, we employed a device structure with *λ*^SW^ = 12 μm and *d* = 20 μm for all the measurements; here, *λ*^SW^ and *d* are the period of a CPW pattern and the gap distance between two CPWs as shown in Fig. 1c and Supplementary Fig. 3a, respectively. The wave number distribution of the induced spin excitations is determined by the Fourier transform of CPW pattern (Supplementary Fig. 2)^19^, whose maximum intensity is always located at *k*^SW^ = 2*π*∕*λ*^SW^ ≈ 0.5 μm^−1^. The propagation direction of *k*^SW^ and *H* are set parallel to the [110] direction of the Cu_2_OSeO_3_ crystal.

Figure 1d indicates the *H*–*T* magnetic phase diagram for Cu_2_OSeO_3_, obtained for the field cooling path shown by the arrows. Although the SkX phase usually appears as the thermodynamic equilibrium state only for the narrow temperature range just below *T*_c_ (Supplementary Fig. 1)^13,14^, the rapid cooling (≈5 K min^−1^) of the sample at 17.5 mT enabled us to keep the SkX phase as a metastable state^21^ even down to 10 K. The corresponding *H*-dependence of local magnetic resonance spectra *ΔL*_11_ measured at 25 K is plotted in Fig. 1e, where the pure SkX phase characterized by the three magnetic resonance modes^15,16^ is clearly identified between 20 mT and 47.5 mT.

**Fig. 2:**
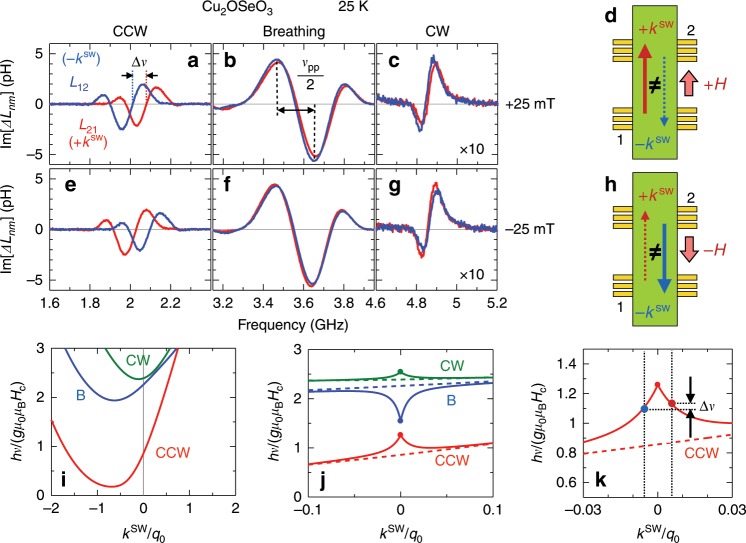
Propagation character of spin excitations on skyrmion strings. **a**–**c** The spectra of mutual inductance *ΔL*_21_ and *ΔL*_12_, which represent the propagation character of spin excitation with the wave vector  +*k*^SW^ and  −*k*^SW^, respectively. The data were measured in the SkX state under the configuration shown in **d** at 25 K with *μ*_0_*H* = +25 mT. **a**, **b**, and **c** represent the CCW, B, and CW modes, respectively. **e**–**h** The corresponding data measured with *μ*_0_*H* = −25 mT. **i**–**k** Dispersion relation for various spin excitations on skyrmion strings, theoretically calculated for the *k*^SW^∥*H* configuration with *H* = 0.4*H*_c_. Here, *h*, *g*, *μ*_0_, *μ*_B_, *H*_c_, and *q*_0_ are Planck constant, *g*-factor, vacuum magnetic permeability, Bohr magneton, critical field value to induce FM state, and magnetic modulation pitch, respectively. In **j** and **k**, the solid and dashed lines represent the dispersion relation for a finite sample in the magnetostatic limit and an infinitely extended sample in the bulk limit, respectively.

Next, we investigated the propagation characteristics of these spin excitations in the SkX phase. Figure 2a–c indicate the spectra of mutual inductance *ΔL*_21_ and *ΔL*_12_ measured at 25 K and +25 mT, which represent the propagation character of spin excitations with the wavevector +*k*^SW^ and −*k*^SW^ (i.e., parallel and antiparallel to the external magnetic field as shown in Fig. 2d), respectively. Here, Fig. 2a–c correspond to the CCW, breathing and CW modes as schematically illustrated in Fig. 1f–h, respectively. For all modes, a propagating signal of coherent spin excitations is observed, implying that the skyrmion strings are well-defined and free of defects over an extended distance. Importantly, for the CCW mode, we can identify a clear frequency shift *Δν* between *ΔL*_21_ and *ΔL*_12_, which demonstrates that the spin excitations propagating along the positive and negative direction on the skyrmion strings are not equivalent. On the other hand, the magnitude of *Δν* is relatively small for the breathing and CW modes. When the direction of external *H* is reversed, the sign of *Δν* is reversed as shown in Fig. 2e–h. Such a non-reciprocal behavior and its mode dependence can be interpreted in terms of the dispersion relation as discussed below.

### Dispersion relation in the SkX state

Figure 2i shows the spin excitation dispersion in the SkX phase, theoretically calculated for the present *k*^SW^∥*H* configuration (see Supplementary Note 2 for details). The dispersion relations are asymmetric (i.e., the resonance frequency is not an even function of *k*^SW^) for all three modes and this asymmetry is most pronounced for the CCW mode. Here, the experimentally measured *Δν* corresponds to the difference of eigen frequencies between *k*^SW^ = ±2*π*∕*λ*^SW^, which directly reflects the degree of asymmetry in the dispersion relation. Therefore, the observation of the largest magnitude of *Δν* in the CCW mode is in accord with the predicted dispersions in Fig. 2i.

In the following, we discuss the microscopic origin of the observed non-reciprocity and its mode dependence. For skyrmion strings, we find that there are two contributions to *Δν*: the first is attributed to the DM interaction, *Δν*_DM_, and the second arises from the dynamic dipolar interaction, *Δν*_dip_. We refer for a discussion of the latter to Supplementary Note 2, and focus here on the contribution from the DM interaction^22–26^. Previously, it has not systematically been understood how DM interaction affects the dispersion relation in the non-collinear magnets with arbitrary excitation modes. As detailed below, we derive a general analytic expression for *Δν*, which reveals that the spatial distribution of the local precession amplitude, as well as the static spin texture in the ground state, has an important role to define the degree of dispersion asymmetry in each mode.

Figure 1f–h represent snapshot images describing how the local spin excitations launched at the *z* = 0 plane propagates on a skyrmion string (aligned along the *H*∥*z* direction) with the wave vector +*k*^SW^ and −*k*^SW^. In the right panel, the corresponding cross-sectional images for selected *z*-planes are also indicated. Such a propagating spin excitation induces a change of the DM energy density *ε*_DM_ = **D**_*z*_(**m**_*i*_ × **m**_*j*_), which is defined between the local moments **m**_*i*_ and **m**_*j*_ on adjacent sites *i* and *j* along the $\hat{z}$-direction with the DM vector ${{\bf{D}}}_{z}=D\hat{z}$. At the edge region of the skyrmion, the local moment is pointing parallel to **H** in the ground state, and precesses CCW around **H** in the excited state as shown in Fig. 1i. In this case, the +*k*^SW^ and −*k*^SW^ excitation modes locally induce a conical spin arrangement along the $\hat{z}$-direction but with opposite sign of the spin helicity (**m**_*i*_ × **m**_*j*_) and thus of *ε*_DM_, which leads to a different excitation energy for modes with  ±*k*^SW^ and causes an asymmetric dispersion. At the core region of the skyrmion, on the other hand, the local moment is antiparallel to **H** and precesses CW around **H**; therefore, the sign of *ε*_DM_ contribution is reversed as compared with the skyrmion edge position (Fig. 1i). As a consequence, the magnitude of non-reciprocity deriving from the DM interaction is basically determined by the imbalance of local precession amplitudes between the edge and core positions with local moments pointing along $+\hat{z}$ and $-\hat{z}$ directions, respectively.

More precisely, *Δν*_DM_ due to the DM interaction in the limit of small *k*^SW^ is up to a normalization given by

1 $$\Delta {\nu }_{{\rm{DM}}}\propto D| {k}^{{\rm{SW}}}| {\int \!}_{{\rm{u.c.}}}{d}^{2}{\bf{r}}\ \hat{z}\ i(\delta {\bf{m}}({\bf{r}})\times \delta {{\bf{m}}}^{* }({\bf{r}}))\propto D| {k}^{{\rm{SW}}}| {\int \!}_{{\rm{u.c.}}}{d}^{2}{\bf{r}}\ {m}_{0}^{z}({\bf{r}}){\mathcal{A}}({\bf{r}}),$$

with the integration range defined by the two-dimensional magnetic unit cell of the SkX. Here, we assume the local magnetization dynamics ${\bf{m}}({\bf{r}},t)={{\bf{m}}}_{0}({\bf{r}})+(\delta {\bf{m}}({\bf{r}})\exp [-i2\pi \nu t]+{\rm{c.c.}})$, with **m**_0_(**r**) and **δm**(**r**) representing the static and dynamical parts of local magnetization component, respectively; ${m}_{0}^{z}({\bf{r}})$ is $\hat{z}$-component of **m**_0_(**r**). The cross product in the integrand is proportional to the local precession intensity ${\mathcal{A}}({\bf{r}})$ as defined in Fig. 1n, i.e., the area enclosed by the precessing local magnetization during a single oscillation period. This allows for an intuitive geometric interpretation of *Δν*_DM_ as given by the last term in equation (1). Its integrand is determined by the product of ${m}_{0}^{z}({\bf{r}})$ and precession density ${\mathcal{A}}({\bf{r}})$ for each excitation mode, whose spatial distribution are presented in Fig. 1j, k–m, respectively. In case of the CCW mode (Fig. 1k), the precession density is confined to the edge of the unit cell, resulting in a large magnitude of *Δν*_DM_ according to equation (1). For the CW mode (Fig. 1m), in contrast, a finite precession density is present both at the core and the edge regions, which leads to a cancelation for *Δν*_DM_. The breathing mode (Fig. 1l) is characterized by a small precession density at the core and the edge region and therefore hosts only a tiny *Δν*_DM_. This analysis accounts already qualitatively for the large *Δν* observed for the CCW mode, and demonstrates that the spatial details of the internal magnetic texture, as well as its excitation manner plays a crucial role for the magnitude of non-reciprocity. As we explain in Supplementary Note 2, for a quantitative comparison an additional contribution *Δν*_dip_ attributed to the dynamical stray field must be taken into account.

The preceding discussion focused on the dispersion relation in the bulk limit *k*^SW^  ≫ 1∕*b*. For *k*^SW^ ≲ 1∕*b*, the magnetostatic (i.e., magnetic dipolar) interaction results in an additional peak structure in the dispersion whose sharpness scales with the sample thickness *b*^27^. Our present experimental setup corresponds to *k*^SW^ ≈ 1∕*b*. As sketched in Fig. 2j, k, this magnetostatic dispersion (solid lines) develops on the background of the non-reciprocal bulk spectrum (dashed lines) so that *Δν* is still dominated by the latter. Nevertheless, the group velocity *v*_g_ = 2*π*(∂*ν*∕∂*k*^SW^) is strongly influenced by the magnetostatic interactions for *k*^SW^ ≲ 1∕*b*. Experimentally, the magnitude of group velocity can be deduced based on the relationship ∣*v*_g_∣ = *ν*_pp_*d*^19^, with *ν*_pp_ representing the oscillation period of *ΔL*_nm_ signals as shown in Fig. 2b.

**Fig. 3:**
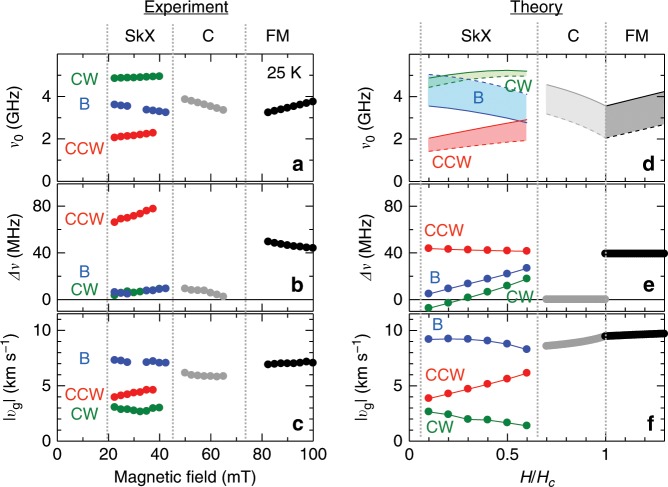
Magnetic field dependence of propagation character for various spin excitation modes. **a**–**c** Experimentally obtained magnetic field dependence of (**a**) magnetic resonance frequency *ν*_0_, (**b**) frequency shift *Δν* between ±*k*^SW^, and (**c**) magnitude of group velocity ∣*v*_g_∣ at 25 K. It is noteworthy that the data for the conical spin state is taken in the *H*-increasing run after the zero field cooling (Supplementary Fig. 1). **d**–**f** The corresponding data calculated from the theoretical dispersion relation. In **d**, the *k*^SW^ → 0 values in the bulk and magnetostatic limit are plotted as dashed and solid lines, respectively, whose difference *δν*_d_ provides an estimate of the group velocity *v*_g_ ≈ 2*πb*(*δν*_d_) shown in **f** with *b* being the sample thickness. In **e**, the non-reciprocal frequency shift *Δν* is shown computed for ∣*k*^SW^∣ = 0.5 μm^−1^.

Figure 3a–c indicate the magnetic field dependence of magnetic resonance frequency *ν*_0_, frequency shift *Δν* between ±*k*^SW^, and magnitude of group velocity ∣*v*_g_∣, experimentally deduced at 25 K from the similar data sets as shown in Fig. 2a–c. Theory provides parameter-free predictions for these quantities that are also plotted in Fig. 3d–f. In both cases, the non-reciprocal frequency shift *Δν* is much larger for the CCW mode than for the other modes, and the magnitude of ∣*v*_g_∣ is the largest and smallest for the breathing and CW modes, respectively. The order of *Δν* and ∣*v*_g_∣ are also roughly in accord with each other. Such a good agreement between the experimental and theoretical results firmly establishes the overall picture of dispersion relations for skyrmion-string excitations.

### Decay length and damping parameter

**Fig. 4:**
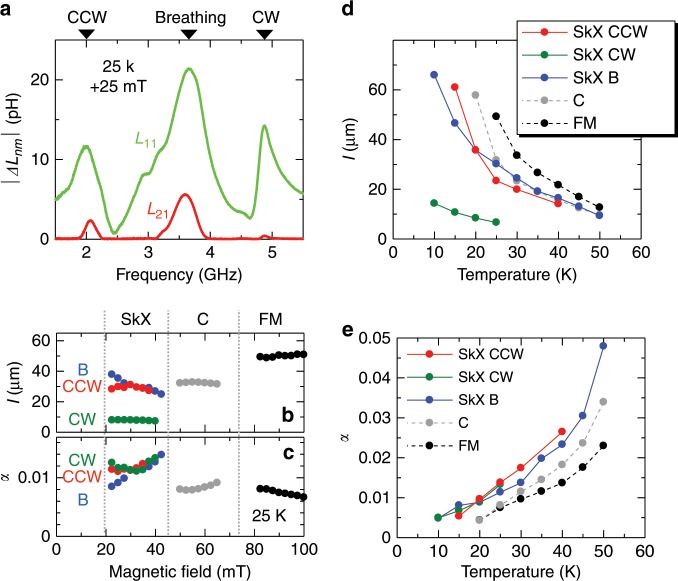
Decay length and damping parameter associated with the propagation of spin excitations. **a** Amplitude spectra of self-inductance ∣*ΔL*_11_∣ and mutual inductance ∣*ΔL*_21_∣ measured in the SkX state at 25 K and +25 mT, whose ratio gives the decay rate of spin excitation amplitude during the propagation. **b**, **c** Magnetic field dependence of decay length *l* and damping parameter *α* measured at 25 K. **d**, **e** Temperature dependence of decay length and damping parameter for various spin excitation modes.

On the basis of the present experimental data, we can further deduce the decay length *l* of propagating spin excitations. Figure 4a indicates the amplitude spectra of self-inductance ∣*ΔL*_11_∣ and mutual inductance ∣*ΔL*_21_∣ measured at 25 K and +25  mT, whose ratio provides the decay rate of spin excitation amplitude during the propagation over the distance *d*. The decay length *l* can be estimated from the relationship $2| \Delta {L}_{21}| /| \Delta {L}_{11}| =\exp (-d/l)$, which is associated with the damping parameter *α* in the form of *l* = *v*_g_∕(2*πν*_0_*α*)^19^. In Fig. 4b, c, the magnetic field dependence of *l* and *α* at 25 K are plotted. Although the decay length *l *largely depends on the character of spin excitation modes, basically the same value of *α* is shared by all three modes in the SkX phase. Here, the CW mode hosts considerably shorter *l *than the other two modes, reflecting its large *ν*_0_ and small *v*_g_ values (Fig. 3a, c). In Fig. 4d, e, temperature dependence of *l* and *α* for various spin excitation modes are plotted. For all temperatures, the SkX phase is characterized by a damping parameter *α* that is slightly larger, but less than a factor of two, than the one in the field-induced ferromagnetic phase. Here, the effective damping parameter *α* reflects not only the intrinsic Gilbert damping, but also the additional contribution of extrinsic origin. In the present case, this slight enhancement of *α* might arise from the elliptical nature of local magnetization precession^28^ and/or the additional scattering, e.g., from defects of the SkX order. Recently, it was suggested that the SkX contains a considerable amount of singular point defects interpreted as emergent magnetic monopoles^5^, which act like the slider of a zipper connecting two skyrmion strings. The existence of such monopole-like defects diminishes the spatial long-range order of the SkX and thus also the coherent propagation of spin excitation along the skyrmion strings. Nevertheless, the decay length *l* in the SkX phase still exceeds 50 μm at low temperatures, proving that the spin excitations on the skyrmion strings can propagate a distance exceeding 10^3^ times the diameter of single skyrmion string (*a*_sk_ ≈ 50 nm^13,14^). This large *l*∕*a*_sk_ ratio yields a lower bound characterizing the distance between defects, and demonstrates the excellent static long-range order of the magnetic skyrmion crystal. Such a small damping and long propagation distance of the excitation are rather unexpected for the SkX phase with its intricate three-dimensional spin texture and the above results suggest that the skyrmion string with excellent long-range order can be a good medium for magnon transport.

## Discussion

In this study, we experimentally demonstrated the coherent and non-reciprocal propagation of spin excitations along skyrmion strings. Unlike conventional ferromagnets, skyrmion strings host multiple excitation modes, and their group velocity, decay length, and degree of non-reciprocity turned out to be strongly mode-dependent in full agreement with our calculated dispersion relations. These results establish the comprehensive picture of the propagation dynamics of skyrmion-string excitations. Moreover, we developed a general theoretical framework to evaluate the dispersion asymmetry for non-collinear magnets, which reveals that the underlying static spin texture and spatial distribution of local precession amplitude are key factors that determine the degree of non-reciprocity for each mode. As the propagating spin excitations carry energy and momentum, the above findings suggest the possibility of unidirectional information transfer along skyrmion strings.

On a broader perspective, the interesting extension of the present work includes the study of the skyrmion-string dynamics under more general conditions. While the current experiments focused on straight skyrmion strings embedded in the skyrmion crystals, the dynamics of a single isolated string has also been discussed theoretically very recently^29–31^. Such skyrmion strings can be bent into curved shapes due to their topological protection^5^ and the spin excitations are expected to propagate along the same string path in analogy with the Kelvin mode of vortex lines in superfluids^7,8,30^. These features suggest that skyrmion strings may serve as robust and flexible information transmission lines potentially enabling the design of associated network or circuits without Joule heat loss, in a similar manner as the recently proposed reconfigurable spin-wave nanochannels^32^. The nonlinear dynamics of these strings is another fascinating issue. Our results highlight that not only the skyrmion particle in two-dimensional systems, but also the skyrmion string in three-dimensional systems is an attractive topological object as a building block for energy-efficient spintronic devices and further investigation of its fundamental and functional properties will be a promising endeavor.

## Methods

### Crystal growth and device fabrication

Single crystals of Cu_2_OSeO_3_ were grown by the chemical vapor transport method. A pair of Au coplanar waveguides (CPW: 200 nm thickness) were fabricated on the oxidized silicon substrate through the standard photolithography technique, and a plate-shaped single crystal of Cu_2_OSeO_3_ (~2 μm thickness) was placed across them with W (tungsten) deposition at an edge of the crystal using the focused ion beam micro fabrication technique (Fig. 1b).

### Propagating spin-wave spectroscopy

By measuring the magnetic contribution to the complex spectra of self-inductance *ΔL*_11_ and mutual inductance *ΔL*_nm_ (with *m* and *n* being the port number used for the excitation and detection, respectively) for these CPWs with the vector NA, the local excitation character and propagation character of spin excitation can be directly evaluated, respectively^19,20^. The spin excitation contribution to the inductance spectrum $\Delta {L}_{\mathrm{{nm}}}(\nu )={L}_{\mathrm{{nm}}}(\nu )-{L}_{\mathrm{{nm}}}^{{\rm{ref}}}(\nu )$ is derived by the subtraction of the common background ${L}_{\mathrm{{nm}}}^{{\rm{ref}}}(\nu )$ from the raw data *L*_nm_(*ν*). Here, *L*_nm_(*ν*) taken at *μ*_0_*H* = 250 mT is adopted as the reference spectrum ${L}_{\mathrm{{nm}}}^{{\rm{ref}}}(\nu )$, where the magnetic resonance is absent within our target frequency range of 0.2 GHz ≤ *ν* ≤ 7.0 GHz. Through this background subtraction process, the possible contribution of cross-talk can be safely excluded. Unless specified, we employed the device with the central wave length *λ*^SW^ = 12 μm and the gap distance *d* = 20 μm.

## Supplementary information

Supplementary Information

## Data Availability

The data presented in the current study are available from the corresponding authors on reasonable request.
