# Supplementary material for: Propagation dynamics of spin excitations along skyrmion strings
Source: Nat Commun. 2020 Jan 14;11:256. doi: 10.1038/s41467-019-14095-0 (PMC6959257; doi:10.1038/s41467-019-14095-0)
Supplement: Supplementary file 1 — Supplementary Information [file 41467_2019_14095_MOESM1_ESM.pdf]

Supplementary information:

Propagation dynamics of spin excitations along skyrmion strings

S. Seki[1,2,3,4], M. Garst[5,6], J. Waizner[7], R. Takagi[1,2,3], N. D. Khanh[3], Y. Okamura[1], K. Kondou[3], F. Kagawa[1,3], Y. Otani[3,8], and Y. Tokura[1,3]

[1] *Department of Applied Physics, University of Tokyo, Tokyo 113-8656, Japan,*

[2] *Institute of Engineering Innovation, University of Tokyo, Tokyo 113-8656, Japan,*

[3] *RIKEN Center for Emergent Matter Science (CEMS), Wako 351-0198, Japan,*

[4] *PRESTO, Japan Science and Technology Agency (JST), Kawaguchi 332-0012, Japan,*

[5] *Institut für Theoretische Physik, Technische Universität Dresden, 01062 Dresden, Germany,*

[6] *Institut für Theoretische Festkörperphysik, Karlsruher Institut für Technologie, 76131 Karlsruhe, Germany,*

[7] *Institut für Theoretische Physik, Universität zu Köln, Zülpicher Str. 77a, 50937 Köln, Germany,*

[8] *Institute for Solid State Physics, University of Tokyo, Kashiwa 277-8581, Japan*

I. SUPPLEMENTARY NOTE 1: EXPERIMENTAL DETAILS

A. Thermodynamic equilibrium phase diagram of Cu_2OSeO_3

Supplementary Fig. 1a indicates H-T magnetic phase diagram for Cu_2OSeO_3, obtained with the zero field cooling procedure followed by the H-increasing scans. This represents the thermodynamic equilibrium phase diagram, where the SkX phase appears only for the narrow T-H region just below T_c[1, 2]. The corresponding H-dependence of magnetic resonance spectra ΔL_{11} measured at 25 K are plotted in Supplementary Fig. 1b. From the comparison with the theoretically predicted behaviors[3], we can identify the pure conical spin state between 30 mT and 70 mT. In Figs. 3a-c in the main text, the data sets for the conical spin state were measured through such a zero field cooling procedure.

B. Dependence on the wave number of spin excitation

In the present study, the CPW pattern as shown in Supplementary Fig. 2a is employed for the generation and detection of spin excitation. The injection of oscillating electric current I^{ν}

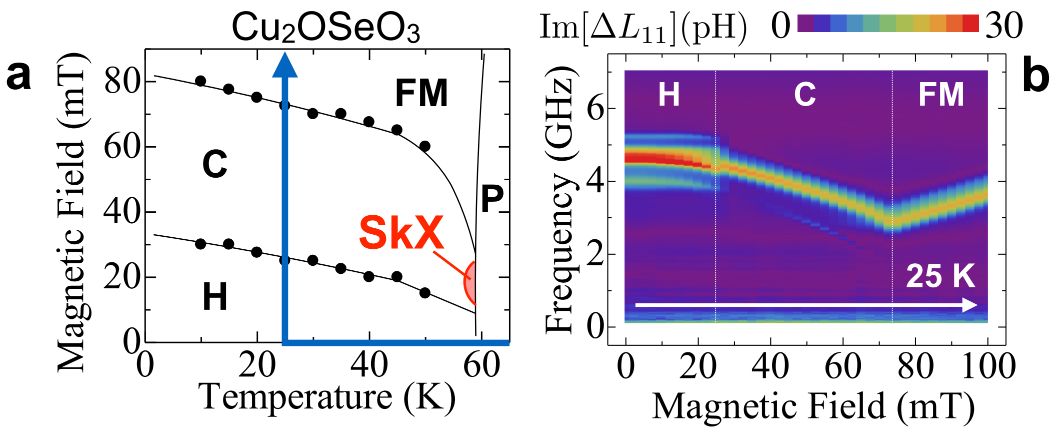

Supplementary Figure 1: **a**, H-T magnetic phase diagram for Cu_2OSeO_3, obtained with the zero field cooling procedure as shown by the arrow. SkX, C, H, FM, and P represent the skyrmion lattice, conical, helical, ferromagnetic, and paramagnetic states, respectively. **b**, The corresponding H-dependence of magnetic resonance spectra ΔL_{11} at 25 K. The data is taken for the H-increasing process after zero field cooling.

into a CPW generates oscillating magnetic field H^ν via the Biot-Savart law, which couples to the spin excitation in the neighboring Cu_2OSeO_3 crystal. Here, the wave number distribution of induced spin excitation is given by the Fourier transform of the spatial distribution of electric current density[4, 5]. In Supplementary Fig. 2b, the corresponding profiles for the CPW pattern in Supplementary Fig. 2a with the period of $\lambda^{\mathrm{SW}} = 12\mu$m and $\lambda^{\mathrm{SW}} = 24\mu$m are plotted. In both cases, the maximum peak intensity appears at $k_{\mathrm{p}}^{\mathrm{SW}} = 2\pi/\lambda^{\mathrm{SW}}$. Since the intensity of higher order peak is almost one order of magnitude smaller, we analyzed our ΔL_{nm} data assuming that the contribution from the main peak centered at $k_{\mathrm{p}}^{\mathrm{SW}}$ is dominant.

Note that Eq. (1) in the main text predicts the relationship $\Delta\nu \propto |k_{\mathrm{p}}^{\mathrm{SW}}|$, which suggests that the magnitude of frequency shift $\Delta\nu$ between $\pm k^{\mathrm{SW}}$ (as observed in Fig. 2a in the main text) should depend on the $|k_{\mathrm{p}}^{\mathrm{SW}}|$ value of the CPW pattern. In Supplementary Fig. 2c, the $\Delta\nu$ value experimentally measured for the CCW mode in the SkX state is plotted as a function of $|k_{\mathrm{p}}^{\mathrm{SW}}|$. This data confirms the predicted $\Delta\nu \propto |k_{\mathrm{p}}^{\mathrm{SW}}|$ relationship, in accord with the asymmetric dispersion as shown in Figs. 2i-k in the main text.

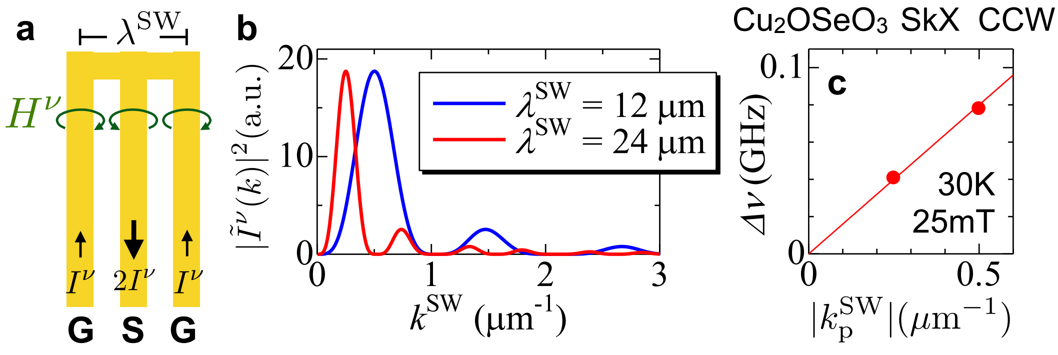

Supplementary Figure 2: **a**, Schematic illustration of coplanar waveguide pattern used for the present study, which consists of a signal (S) line and a pair of ground (G) lines. The oscillating electric current I^ν injected from network analyzer generates oscillating magnetic field H^ν. Here, G and S lines and the space between them have the width of $\lambda^{\mathrm{SW}}/4$. **b**, Fourier transform of the current distribution $|I^\nu(k)|$ for the waveguide pattern shown in **a**, calculated with various λ^{SW} values. Here, the maximum peak intensity always appears at $k_{\mathrm{p}}^{\mathrm{SW}} = 2\pi/\lambda^{\mathrm{SW}}$. **c**, $|k_{\mathrm{p}}^{\mathrm{SW}}|$-dependence of frequency shift $\Delta\nu$ between $\pm k^{\mathrm{SW}}$, measured for the CCW mode in the SkX state at 30K and +25mT using two different waveguide patterns considered in **b**. The definition of $\Delta\nu$ is given in Fig. 2a in the main text.

C. Dependence on the gap distance

In general, the ratio between the self-inductance $|\Delta L_{11}|$ and mutual inductance $|\Delta L_{21}|$ provides the decay rate of spin excitation amplitude during the propagation for the gap distance d between two CPWs used for the excitation and detection[4]. In Supplementary Fig. 3a, the $2|\Delta L_{21}|/|\Delta L_{11}|$ value (deduced from the similar experimental data sets as shown

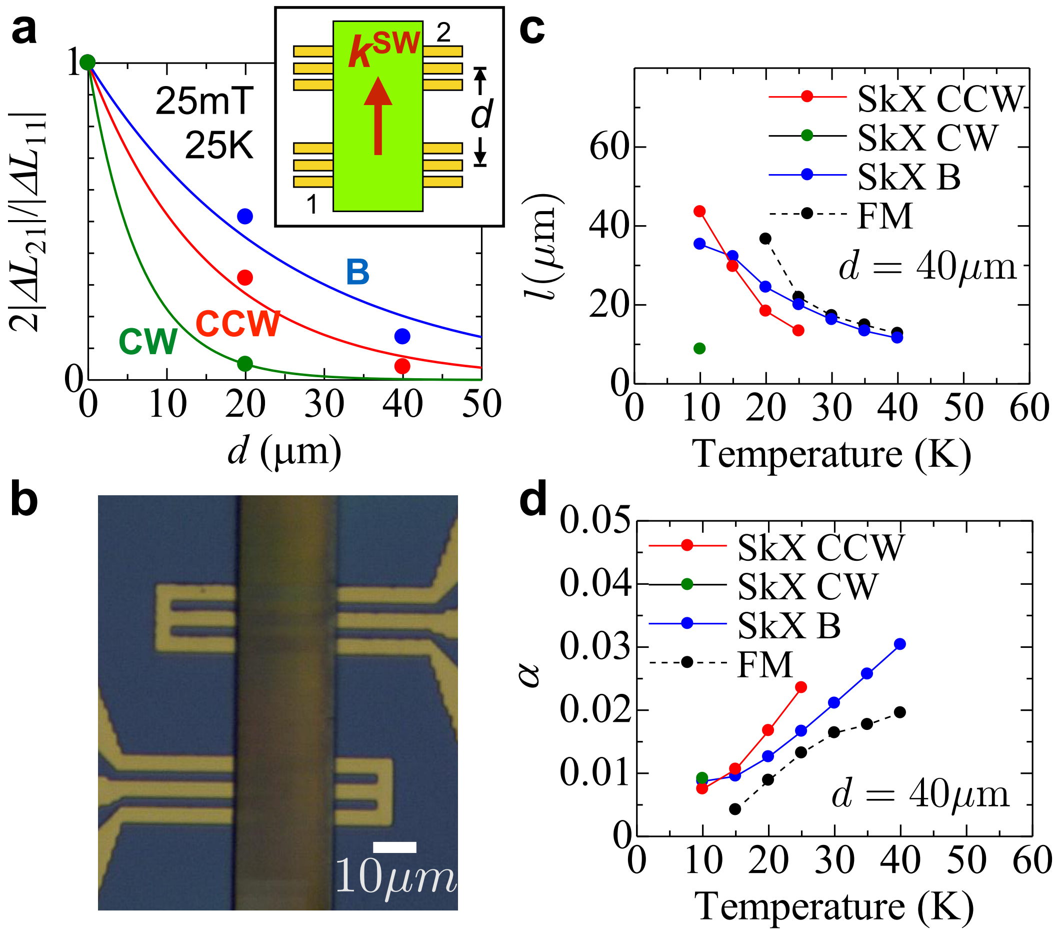

Supplementary Figure 3: **a**, The decay rate of spin excitation amplitude during the propagation, plotted as a function of the gap distance d. The measurement is performed for various spin excitation modes in the SkX state at 25K and 25mT. The corresponding spectra of $|\Delta L_{21}|$ and $|\Delta L_{11}|$ for $d = 20\mu$m are shown in Fig. 4a in the main text. The solid lines represent the theoretical fitting by the exponential decay function $2|\Delta L_{21}|/|\Delta L_{11}| = \exp(-d/l)$. **b**, Top-view optical image of the device with $d = 40\mu$m. **c**,**d**, Temperature dependence of decay length l and damping parameter α, obtained from the device with $d = 40\mu$m.

in Fig. 4a in the main text) is plotted for various excitation modes in the SkX state as a function of d. It roughly follows the expected $2|\Delta L_{21}|/|\Delta L_{11}| = \exp(-d/l)$ relationship[4], where the slight deviation from this curve is mainly ascribed to the device dependence of the unintentional air gap between the CPWs and Cu_2OSeO_3 sample that is inevitable in the FIB micro-fabrication process.

Here, the decay length l is related with the damping parameter α in form of $l = v_g/(2\pi\nu_0\alpha)$[4]. In Supplementary Fig. 3c and d, temperature dependence of l and α for various excitation modes, experimentally deduced for the device with $d = 40\mu$m (Supplementary Fig. 3b), are plotted. The corresponding data for the device with $d = 20\mu$m are also shown in Figs. 4d and e in the main text. In both cases, the SkX phase always hosts slightly larger, but less than twice of, α value as compared to the ferromagnetic phase. The order of l and α values for these two devices also roughly agree with each other. These results confirm the overall reliability of observed mode dependence of l and α. (Note that the high temperature data for the $d = 40\mu$m device become less reliable than the $d = 20\mu$m device, because the $2|\Delta L_{21}|/|\Delta L_{11}|$ value for the former one is more suppressed due to the longer distance of propagation.)

As discussed in the main text, the effective damping parameter α reflects not only the intrinsic Gilbert damping, but also the scattering by the imperfections in the magnetic order such as defects in the skyrmion crystal. Therefore, the only slightly enhanced value of α in the SkX state as compared with the FM state suggests that the defect density in the skyrmion crystal is rather low, which demonstrates the well long-range ordered nature of the skyrmion string structure despite its complex internal spin texture.

Note that the decay length l often exceeds 1mm in case of ferrimagnetic YIG with lower intrinsic Gilbert damping[6–9], and further search of novel material systems allowing longer distance of signal transfer through skyrmion strings would be a future challenge.

D. Dependence on the geometrical configuration

In the main text, we discussed the results for the $H \parallel k^{\mathrm{SW}}$ configuration (Fig. 2d), where the spin excitation propagates parallel to the skyrmion strings in the SkX state. For the $H \perp k^{\mathrm{SW}}$ configuration (Supplementary Fig. 4a), on the other hand, we can investigate the character of spin excitation propagating normal to the skyrmion strings. Supplementary

Figs. 4b and c indicate the spectra of mutual inductance ΔL_{21} and ΔL_{12} (representing the propagation character of spin excitation with the wavevector $+k^{\mathrm{SW}}$ and $-k^{\mathrm{SW}}$) measured with the $H \perp k^{\mathrm{SW}}$ configuration for the CCW and breathing modes in the SkX state (Note that the signal of the CW mode is too weak to be detected here). The corresponding data measured in the ferromagnetic state is also plotted in Supplementary Fig. 4d. Unlike the case of $H \parallel k^{\mathrm{SW}}$, nonreciprocal propagation behavior or associated frequency shift $\Delta\nu$ between $\pm k^{\mathrm{SW}}$ have not been observed for any mode in the $H \perp k^{\mathrm{SW}}$ configuration. Such an absence of nonreciprocity is consistent with symmetry analysis; For the present $H \perp k^{\mathrm{SW}}$ configuration (Supplementary Fig. 4a), the two-fold rotational symmetry around H is sustained, which requires the equivalent nature of $+k^{\mathrm{SW}}$ and $-k^{\mathrm{SW}}$. The above results demonstrate that the appearance or absence of nonreciprocity is strongly dependent on the geometrical relationship between the directions of k^{SW}-vector and skyrmion strings.

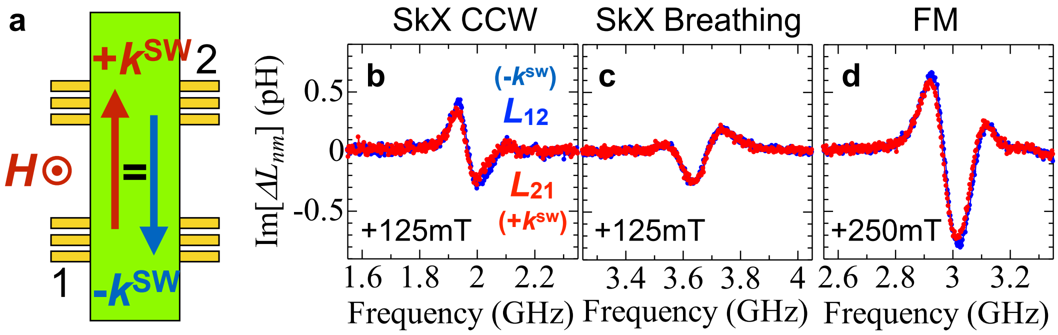

Supplementary Figure 4: **a**, Schematic illustration of the measurement configuration for the $H \perp k^{\mathrm{SW}}$ setup. **b-d**, The spectra of mutual inductance ΔL_{21} and ΔL_{12}, which represent the propagation character of spin excitation with the wave vector $+k^{\mathrm{SW}}$ and $-k^{\mathrm{SW}}$, respectively. All the data were measured at 30 K under the configuration shown in **a**. Here, **b** and **c** represent the CCW and breathing modes in the SkX state at +125 mT, respectively, and **d** indicates the resonance mode in the collinear ferromagnetic state at +250 mT. Note that the H-value required for the stabilization of the SkX phase is different between the $H \perp k^{\mathrm{SW}}$ and $H \parallel k^{\mathrm{SW}}$ configurations, due to the demagnetization effect.

II. SUPPLEMENTARY NOTE 2: THEORETICAL CALCULATIONS

We present details of our theoretical analysis supporting the conclusion in the main text. The computations are performed in the framework of linear spin wave theory for the skyrmion crystal following Supplementary Refs. 3 and 10. We present in section II A the energy functional for chiral magnets and the wave equation for the spin excitations. We also specify the parameters entering the theory and explain the connection to experiment. In sections II B and II C we discuss the field-polarized and conical phase, respectively. The numerical solution for the skyrmion crystal phase is explained in section II D. The section II E focusses on the non-reciprocity of spin waves in the skyrmion crystal phase. It is demonstrated that it derives both from the DM interaction and the stray field energy with the main results given in Supplementary Equations (27) and (29).

A. Linear spin wave theory for cubic chiral magnets

1. Free energy and equation of motion

The magnetic properties of the cubic chiral magnets deep within the order magnetic phases are well described in terms of a unit vector $\mathbf{m}(\mathbf{r})$ representing the direction of the magnetization. It is governed by the free energy functional $\mathcal{F} = \mathcal{F}_0 + \mathcal{F}_{\text{dip}}$ with

$$\mathcal{F}_0 = \int d\mathbf{r}\Big[\frac{J}{2}(\nabla_j \mathbf{m}_i)^2 + D\mathbf{m}(\nabla \times \mathbf{m}) - \mu_0 M_s H \mathbf{m}_z + \lambda(\mathbf{m}^2 - 1)^2\Big] \tag{1}$$

where J is the exchange interaction, D is the DM interaction, M_s is the saturation magnetization, μ_0 is the magnetic constant, and H is the magnetic field applied along the z-axis. In the following, we assume a right-handed magnetic system with positive $D > 0$. It is convenient to impose the condition $\mathbf{m}^2(\mathbf{r}) = 1$ approximately with the help of a 'soft-spin' implementation represented by the last term in Supplementary Equation (1). In the following, we use a fixed $\lambda = 160000$ so that the length $|\mathbf{m}(\mathbf{r})|$ varies, for example, by less than half per mille within the skyrmion crystal phase.

The stray field energy reads

$$\mathcal{F}_{\text{dip}} = \int \frac{d\mathbf{k}}{(2\pi)^3}\frac{1}{2}\mathbf{m}_i(-\mathbf{k})\chi^{-1}_{\text{dip},ij}(\mathbf{k})\mathbf{m}_j(\mathbf{k}) \tag{2}$$

with the Fourier transform $\mathbf{m}(\mathbf{k}) = \int d\mathbf{r} e^{-i\mathbf{k}\mathbf{r}} \mathbf{m}(\mathbf{r})$. For wavevectors much larger than the inverse linear size of the sample $|\mathbf{k}| \gg 1/L$, the susceptibility is given by $\chi^{-1}_{\text{dip},ij}(\mathbf{k}) = \mu_0 M_s^2 \frac{\mathbf{k}_i \mathbf{k}_j}{\mathbf{k}^2}$. For zero wavevector it is determined by the demagnetization factors $\chi^{-1}_{\text{dip},ij}(0) = \mu_0 M_s^2 N_{ij}$ with $N_{ij} = \text{diag}(N_x, N_y, N_z)$ for an ellipsoidal sample. For intermediate wavevectors $|\mathbf{k}|L \lesssim 1$, the system is in the magnetostatic limit where the magnetic properties depend on the details of the sample size.

The equation of motion that governs the magnetization dynamics is given by

$$\partial_t \mathbf{m} = -\gamma \mathbf{m} \times \mathbf{B}_{\text{eff}} \tag{3}$$

with the effective field $\mathbf{B}_{\text{eff}} = -\frac{1}{M_s} \frac{\delta \mathcal{F}}{\delta \mathbf{m}}$ and the gyromagnetic ratio $\gamma = g\mu_B/\hbar$. The magnetic phases are identified in equilibrium by a vanishing effective magnetic field $\mathbf{B}_{\text{eff}}|_{\mathbf{m}_0} = 0$ when evaluated with the equilibrium magnetization $\mathbf{m}_0(\mathbf{r})$. In first order in the deviation $\delta\mathbf{m}(\mathbf{r}, t) = \mathbf{m}(\mathbf{r}, t) - \mathbf{m}_0(\mathbf{r})$ the field $\mathbf{B}_{\text{eff},i}(\mathbf{r}, t) = -\frac{1}{M_s} \int d\mathbf{r}' \chi^{-1}_{ij}(\mathbf{r}, \mathbf{r}') \delta\mathbf{m}_j(\mathbf{r}', t)$ is then given in terms of the susceptibility $\chi^{-1}_{ij}(\mathbf{r}, \mathbf{r}') = \delta^2 \mathcal{F}/(\delta\mathbf{m}_i(\mathbf{r})\delta\mathbf{m}_j(\mathbf{r}'))|_{\mathbf{m}_0}$ that is to be evaluated with the equilibrium magnetization $\mathbf{m}_0(\mathbf{r})$. The linear spin wave theory is obtained by expanding the equation of motion (3) in first order in $\delta\mathbf{m}$,

$$\partial_t \delta\mathbf{m}(\mathbf{r}, t) = \frac{\gamma}{M_s} \mathbf{m}_0(\mathbf{r}) \times \int d\mathbf{r}' \chi^{-1}(\mathbf{r}, \mathbf{r}') \delta\mathbf{m}(\mathbf{r}', t) \tag{4}$$

The spin wave spectrum and its eigenvectors are obtained by solving the spin wave equation (4).

2. Parameters entering the theory

Important parameters are the wavevector $Q = D/J$, the internal critical field $\mu_0 H^{\text{int}}_{c2} = D^2/(JM_s)$ separating the conical from the field-polarized phase, and the susceptibility in the conical phase $\chi^{\text{int}}_{\text{con}} = \frac{\mu_0 M_s^2}{JQ^2}$. For Cu_2OSeO_3 we use $Q = 2\pi/\lambda$ with $\lambda = 60$ nm and $\chi^{\text{int}}_{\text{con}} = 1.76$ [3]. The critical field H^{int}_{c2} is temperature dependent and for $T = 25$ K we have $\mu_0 H^{\text{int}}_{c2} \approx 0.07$ T. After measuring length in units of $1/Q$ and energy in units of $g\mu_B \mu_0 H^{\text{int}}_{c2}$ with g factor $g \approx 2.1$, the theory then only depends on the parameters given by the demagnetization factors of the sample and the ratio H/H_{c2} measuring the strength of the applied magnetic field with $H_{c2} = H^{\text{int}}_{c2}(1 + N_z \chi^{\text{int}}_{\text{con}})$. In the present study, the investigated plate-shape sample of Cu_2OSeO_3 can be approximately characterized by the demagnetization

factors:

$$N_x = 0.879, \quad N_y = 0.105, \quad N_z = 0.016, \tag{5}$$

where the magnetic field is applied along the z-axis. In the following discussion, we will also use the abbreviation $\mathcal{D} = g\mu_B\mu_0 H_{c2}^{\rm int}/Q^2$ for the stiffness.

3. Limits of the spin wave dispersion and experimental quantities

In the following, we discuss volume spin waves and their dispersion $\omega(\mathbf{k}) = 2\pi\nu(\mathbf{k})$. We distinguish between the bulk spin wave dispersion for wavevectors $|\mathbf{k}|L \gg 1$ and the magnetostatic limit of the spin wave dispersion for wavevectors $|\mathbf{k}|L \lesssim 1$ with the linear size L of the sample. It is important to note that the limits of small wavevectors $\mathbf{k} \to 0$ and a large bulk sample $L \to \infty$ do not commute. The precise form of the dispersion in the magnetostatic limit depends on the details of the sample shape and the boundary conditions, and we will not attempt to provide a discussion of the dispersion in this regime. We will limit ourselves to a discussion of the bulk dispersion for $|\mathbf{k}|L \gg 1$ and the uniform resonance frequency at zero wavevector, $\nu(0)$. In particular, we focus on the bulk spin wave dispersion for wavevectors longitudinal to the applied magnetic field $\mathbf{H} = H\hat{z}$ that is non-reciprocal $\omega(k_z) \neq \omega(-k_z)$ in chiral magnets and gives access to the following quantities

$$\nu_\infty \equiv \lim_{k_z\to 0}\lim_{L\to\infty}\frac{\omega(k_z)}{2\pi}, \quad v_\infty \equiv \lim_{k_z\to 0}\lim_{L\to\infty}\partial_{k_z}\omega(k_z). \tag{6}$$

The limit ν_∞ and the uniform resonance frequency $\nu(0)$ differ, and the dispersion $\nu(k_z)$ interpolates between these two values in the magnetostatic limit $|k_z|L \lesssim 1$ as sketched, e.g. in Fig. 2j of the main text. Magnetostatic modes with $\nu(0) > \nu_\infty$ and $\nu(0) < \nu_\infty$ are known, respectively, as backward (BVMSW) and forward (FVMSW) volume magnetostatic spin wave modes. The two values ν_∞ and $\nu(0)$ are shown in Fig. 3d of the main text as dashed and solid lines, respectively. The experiment is performed in the magnetostatic limit (as $|k^{\rm SW}|b \sim 1$ in our setup) so that the recorded frequency $\nu(k_z)$, also denoted by ν_0 in the main text, is located within the frequency range enclosed by ν_∞ and $\nu(0)$. As the interpolation between these two values occurs on the scale of a wavevector given by the inverse thickness of the sample $1/b$, we can crudely estimate the experimentally measured group velocity by $v_g \approx 2\pi(\nu(0) - \nu_\infty)b$.

The velocity v_∞ in Supplementary Equation (6) is a measure of the non-reciprocity of the bulk spectrum. We assume that it also determines the non-reciprocity in the magnetostatic limit (with the conical phase being an exception, see below) so that we approximate $\Delta\nu(k_z) = \nu(k_z) - \nu(-k_z) \approx 2v_\infty k_z/(2\pi)$.

We summarize the relation between the measured quantities $\nu_0 = \nu(k_z), \Delta\nu(k_z)$ and $v_g(k_z)$ shown in Fig. 3 of the main text and the theoretically accessible parameters $\nu(0), \nu_\infty$ and v_∞,

$$\nu_0 \in \{\min\{\nu_\infty, \nu(0)\}, \max\{\nu_\infty, \nu(0)\}\}, \tag{7}$$

$$\Delta\nu = \nu(k_z) - \nu(-k_z) \approx \frac{2v_\infty k_z}{2\pi}, \tag{8}$$

$$v_g \approx 2\pi(\nu(0) - \nu_\infty)b. \tag{9}$$

Here, the wavevector k_z denoted by k^{SW} in the main text is assumed to be in the magnetostatic limit $|k^{\mathrm{SW}}|b \sim 1$ with the width b of the sample. These approximations allow for a comparison with theory without the computation of the full dispersion in the magnetostatic limit, which is a formidable task and requires much more effort. Note that all parameters entering the calculation, see section II A 2, are known from independent measurements so that the theory provides quantitative parameter-free predictions for $\nu(0), \nu_\infty$ and v_∞.

In the next sections, we discuss the theoretical values $\nu(0), \nu_\infty$ and v_∞ for the various magnetic phases.

B. Field-polarized phase

In the field-polarized (FP) phase at $H > H_{c2}$, the magnetic ground state within the bulk of the sample is polarized along the applied field, $\mathbf{m}_0 = \hat{H} = \hat{z}$. The bulk spin wave spectrum for a wavevector aligned with the magnetic field is given by [11]

$$\hbar\omega(k_z) = 2\mathcal{D}Qk_z + \mathcal{D}k_z^2 + g\mu_B\mu_0 H_{\mathrm{int}} \tag{10}$$

with the internal field $H_{\mathrm{int}} = H - N_z M_s$. We obtain for the quantities of Supplementary Equations (6)

$$\nu_{\mathrm{FP},\infty} = \frac{g\mu_B\mu_0 H_{\mathrm{int}}}{2\pi\hbar}, \quad v_{\mathrm{FP},\infty} = \frac{2\mathcal{D}Q}{\hbar} = 2\frac{g\mu_B\mu_0 H_{c2}^{\mathrm{int}}}{\hbar Q}. \tag{11}$$

The excitation energy at strictly zero wavevector $\mathbf{k} = 0$ is given by the Kittel formula

$$\nu_{\rm FP}(0) = \frac{g\mu_B\mu_0}{2\pi\hbar}\sqrt{(H-(N_z-N_x)M_s)(H-(N_z-N_y)M_s)}. \quad (12)$$

In the geometry (5) the Kittel frequency is larger than $\nu_{\rm FP,\infty}$ so that the spin wave is a BVMSW with a dynamic magnetization that oscillates within the plane perpendicular to the wavevector $\mathbf{k} = k_z\hat{z}$.

C. Conical phase

The magnetization of the conical phase for $H < H_{\rm c2}$ is given by $\mathbf{m}_0(z) = (\sin\theta\cos Qz, \sin\theta\sin Qz, \cos\theta)$ with the cone angle θ that obeys $M_s\cos\theta = \chi^{\rm int}_{\rm con}H_{\rm int}$. The periodicity of the magnetization leads to Bragg scattering of spin waves and a magnon band structure [11]. The bulk spin wave spectrum for $\mathbf{k} = k_z\hat{z}$ in the extended zone scheme is given by

$$\hbar\omega(k_z) = \mathcal{D}|k_z|\sqrt{k_z^2 + (1+\chi^{\rm int}_{\rm con})Q^2\left(1-\left(\frac{H_{\rm int}}{H^{\rm int}_{c2}}\right)^2\right)}. \quad (13)$$

There are two magnetic resonances in the conical phase denoted by $+Q$ and $-Q$ whose limit ν_∞ for small wavevectors $k_z \to 0$ is degenerate in the repeated zone scheme, and it is obtained by the taking the limit $k_z \to \pm Q$ of Supplementary Equation (13),

$$\nu_{\pm Q,\infty} = \frac{\omega(\pm Q)}{2\pi} = \frac{g\mu_B\mu_0 H^{\rm int}_{c2}}{2\pi\hbar}\sqrt{1+(1+\chi^{\rm int}_{\rm con})\left(1-\left(\frac{H_{\rm int}}{H^{\rm int}_{c2}}\right)^2\right)}. \quad (14)$$

The computation of the group velocity of the bulk spectrum v_∞ is tricky due to the degeneracy $\nu_{\pm Q,\infty}$. Considering the derivative of Supplementary Equation (13) in the limit $k_z \to \pm Q$, one finds a finite velocity with $v_{+Q,\infty} = -v_{-Q,\infty}$, i.e., the dispersion of the $\pm Q$ modes cross at $\nu_{\pm Q,\infty}$. This crossing becomes however an avoided crossing in the magnetostatic limit where the stray field lifts the degeneracy. For this reason, we argue that in the conical phase the equation (8) is not applicable, and instead the non-reciprocity is practically vanishing $\Delta\nu \approx 0$.

We note however that higher-order gradient corrections to the theory of Supplementary Equation (1) can shift the crossing point of the bulk spectrum at energies $\nu_{\pm Q,\infty}$ away from $k_z = 0$ in the repeated zone scheme, see the discussion in Supplementary Refs. [12, 13]. This

leads to a small non-reciprocity even in the conical phase that decreases with the applied magnetic field. This effect is neglected in the theoretical figure of Fig. 3 in the main text.

The uniform resonance frequencies of the two modes $\nu_{\pm Q}(0)$ are known in closed form and given in Supplementary Refs. [3, 11] so that we do not repeat them here. Both modes are BVMSW, $\nu_{\pm Q}(0) > \nu_{\pm Q,\infty}$, as their mean magnetization oscillates within the plane perpendicular to the wavevector $\mathbf{k} = k_z \hat{z}$. However, the spectral weight of the $-Q$ mode is much smaller in the investigated field range [11] so that only the $+Q$ mode is detected in the experiment. Consequently, only the theoretical results for the $+Q$ mode is presented in Fig. 3 of the main text.

D. Skyrmion crystal phase – numerical solution of the spin wave spectrum

1. *Variational Ansatz and spin wave equation*

In order to obtain the magnetization of the skyrmion crystal, we use the variational Ansatz

$$\mathbf{m}_0(\mathbf{r}) = \sum_{\mathbf{G}_\perp \in L_R} \mathbf{m}_0(\mathbf{G}_\perp) e^{i\mathbf{G}_\perp \mathbf{r}} \tag{15}$$

with the Fourier components $\mathbf{m}_0(\mathbf{G}_\perp)$ where the vectors $\mathbf{G}_\perp$ belong to the two-dimensional triangular reciprocal lattice L_R that is perpendicular to the applied magnetic field, $\mathbf{G}_\perp \hat{z} = 0$. In practice, the reciprocal lattice is restricted to a finite number of primitive unit cells of the reciprocal lattice and the symmetries of the skyrmion crystal are exploited in order to reduce the amount of variational parameters $\mathbf{m}_0(\mathbf{G}_\perp)$, for details see Supplementary Ref. [10]. First, the free energy is minimized with the Ansatz (15) and, in a second step, the spin wave equation (4) is solved.

With the help of the Fourier transforms $\delta\mathbf{m}(\mathbf{r}, t) = \int \frac{d\mathbf{q}}{(2\pi)^3} \frac{d\omega}{2\pi} e^{-i\omega t + i\mathbf{q}\mathbf{r}} \delta\mathbf{m}(\mathbf{q}, \omega)$ and $\chi^{-1}(\mathbf{r}, \mathbf{r}') = \int \frac{d\mathbf{q}}{(2\pi)^3} \frac{d\mathbf{q}'}{(2\pi)^3} e^{i\mathbf{q}\mathbf{r} + i\mathbf{q}'\mathbf{r}'} \chi^{-1}(\mathbf{q}, \mathbf{q}')$ this wave equation can be expressed as

$$-i\omega \delta\mathbf{m}(\mathbf{q}, \omega) = \frac{\gamma}{M_s} \sum_{\mathbf{G}_\perp \in L_R} \int \frac{d\mathbf{q}'}{(2\pi)^3} \mathbf{m}_0(\mathbf{G}_\perp) \times \left(\chi^{-1}(\mathbf{q} - \mathbf{G}_\perp, -\mathbf{q}') \delta\mathbf{m}(\mathbf{q}', \omega) \right). \tag{16}$$

Decomposing the wavevectors $\mathbf{q} = \mathbf{K}_\perp + \mathbf{k}$ and $\mathbf{q}' = \mathbf{K}'_\perp + \mathbf{k}'$ into reciprocal lattice vectors, $\mathbf{K}_\perp$ and $\mathbf{K}'_\perp$, and wavevectors $\mathbf{k}$ and $\mathbf{k}'$ whose components perpendicular to the z-axis belong to the first Brillouin zone, $\mathbf{k}_\perp, \mathbf{k}'_\perp \in 1.\text{BZ}$, we can exploit that the susceptibility is diagonal

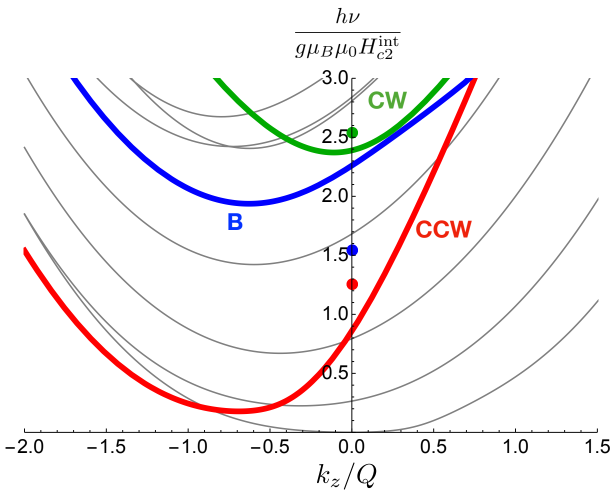

Supplementary Figure 5: The bulk spin wave spectrum $\nu(k_z) = \omega(k_z)/(2\pi)$ for the skyrmion crystal in Cu_2OSeO_3 numerically evaluated for a magnetic field $H/H_{c2} = 0.4$ as a function of wavevector k_z parallel to the applied magnetic field. There are various modes (grey) but only the CCW (red), B (blue) and CW (green) modes possess a global dynamic magnetization. The uniform resonance frequency $\nu(0)$ at $k_z = 0$ of these modes for the geometry of Supplementary Equation (5) is also indicated by the dots.

in wavevectors but only up to reciprocal lattice vectors, $\chi^{-1}_{ij}(\mathbf{K}_\perp + \mathbf{k} - \mathbf{G}_\perp, -\mathbf{K'}_\perp - \mathbf{k'}) = \chi^{-1}_{ij}(\mathbf{K}_\perp - \mathbf{G}_\perp, -\mathbf{K'}_\perp; \mathbf{k})(2\pi)^3\delta(\mathbf{k} - \mathbf{k'})$, so that the wave equation simplifies to

$$\omega\delta\mathbf{m}(\mathbf{K}_\perp + \mathbf{k}, \omega) = \sum_{\mathbf{K'}_\perp \in L_R} \mathcal{W}(\mathbf{K}_\perp, \mathbf{K'}_\perp; \mathbf{k})\delta\mathbf{m}(\mathbf{K'}_\perp + \mathbf{k}, \omega) \tag{17}$$

with the matrix

$$\mathcal{W}_{nm}(\mathbf{K}_\perp, \mathbf{K'}_\perp; \mathbf{k}) = i\frac{\gamma}{M_s} \sum_{\mathbf{G}_\perp \in L_R} \epsilon_{n\ell j}\mathbf{m}_{0,\ell}(\mathbf{G}_\perp)\chi^{-1}_{jm}(\mathbf{K}_\perp - \mathbf{G}_\perp, -\mathbf{K'}_\perp; \mathbf{k}). \tag{18}$$

The solution for a given frequency ω and wavevector $\mathbf{k}$ yields the dispersion $\omega(\mathbf{k})$ and the eigenvectors $\delta\mathbf{m}(\mathbf{K}_\perp + \mathbf{k}, \omega)$.

2. Numerical solution for the spin wave spectrum

The numerical solution for the bulk spin wave spectrum for wavevectors perpendicular to the applied field, $\omega(\mathbf{k}_\perp)$, was presented, e.g., in Supplementary Ref. [11]. Here, we discuss the spectrum for wavevectors along the field $\omega(k_z)$. In Supplementary Fig. 5 the spectrum

numerically evaluated for a magnetic field $H = 0.4H_{c2}$ is shown. There are various modes but only three of them possess a global dynamic magnetization, i.e., an oscillating magnetic dipole moment on average: the counterclockwise (CCW), the breathing (B) and the clockwise (CW) mode indicated by the colored lines. Their dispersion is reproduced in Fig. 2i of the main text. The uniform resonance frequency $\nu(0)$ of these modes at zero wavevector is represented by the colored dots for the geometry of Supplementary Equation (5). We can conclude that the CCW and the CW modes are BVMSW modes as their $\nu_\infty < \nu(0)$ whereas the breathing mode is a FVMSW mode with $\nu_\infty > \nu(0)$. This is consistent with the observation that their global dynamic magnetization oscillates within the plane perpendicular to the wavevector $\mathbf{k} \parallel \mathbf{H}$ for the CCW and CW modes (in a counterclockwise and clockwise manner, respectively), and it is linearly polarized along the wavevector $\mathbf{k} \parallel \mathbf{H}$ for the B mode. The numerically computed values of $\nu(0)$ and ν_∞ for the three modes at various magnetic fields are shown in Fig. 3d of the main text by the solid and dashed lines, respectively.

Moreover, the slope v_∞ of the spectra in Supplementary Fig. 5 close to zero wavevector, that is a measure for the non-reciprocity, is the largest for the CCW mode. In the next section, we present an analytical expression for this slope v_∞ in terms of the spin wave function that elucidates the different non-reciprocities present in Supplementary Fig. 5.

It is instructive to compare the spectrum in Supplementary Fig. 5 of the skyrmion crystal with the excitation spectrum of a single skyrmion string, which was recently studied in Supplementary Refs. [14–16]. The skyrmion crystal is periodic within the plane perpendicular to the applied field, so that its spectrum at the center of the Brillouin zone is discrete due to the finite extension of its Wigner-Seitz cell. As a function of wavevector k_z, this gives rise to distinct dispersive modes. In contrast, a single skyrmion string acts only as an isolated scattering center for spin waves. Consequently, one can distinguish between delocalized scattering states and localized states that are bound to the skyrmion. The former give rise to a continuous spectrum, and the localized bound states yield a discrete spectrum. Similarly to the skyrmion crystal, the dispersion of both the scattering and localized states is non-reciprocal, i.e., asymmetric with respect to the wavevector k_z along the skyrmion string.

E. Skyrmion crystal phase – non-reciprocity of the bulk spin wave spectrum

We aim to derive an analytical expression for the slope v_∞ of the bulk spin wave dispersions for the skyrmion crystal that quantifies their non-reciprocity. When we consider the limit $k_z \to 0$ in the following, it is implied that the limit $L \to \infty$ for an infinitely large sample was taken first in order to comply with the definition of Supplementary Equation (6)

1. *Solution of the spin wave equation for* $\mathbf{k}_\perp = 0$ *and* $k_z \to 0$

In this limit the spin wave equation (17) reads

$$\omega\delta\mathbf{m}(\mathbf{K}_\perp, \omega) = \sum_{\mathbf{K}'_\perp \in L_R} \mathcal{W}(\mathbf{K}_\perp, \mathbf{K}'_\perp; 0\hat{z})\delta\mathbf{m}(\mathbf{K}'_\perp, \omega), \tag{19}$$

where $0\hat{z}$ in the last argument of the matrix $\mathcal{W}$ defined in Supplementary Equation (18) is a reminder of the particular limit we are considering. The eigenvectors with eigenfrequency ω_α will be denoted by $\delta\mathbf{m}_\alpha(\mathbf{K}_\perp)$. These eigenfrequencies identify ν_∞, see Supplementary Equation (6), of the mode with quantum number α. Note that the matrix $\mathcal{W}$ is non-hermitian so that the system must be solved with a Bogoliubov transformation instead of a unitary transformation. As a consequence, the eigenvectors (for positive eigenvalues) are orthonormal with respect to the scalar product

$$\langle \delta m_{\alpha'} | \delta m_\beta \rangle = \sum_{\mathbf{G}_\perp, \mathbf{G}'_\perp \in L_R} \delta\mathbf{m}^\dagger_{\alpha'}(\mathbf{G}'_\perp) i \left(\mathbf{m}_0(\mathbf{G}'_\perp - \mathbf{G}_\perp) \times \delta\mathbf{m}_\alpha(\mathbf{G}_\perp)\right) = \delta_{\alpha,\alpha'}. \tag{20}$$

It is convenient to express this relation in real space with the help of the Fourier transform $\delta\mathbf{m}_\alpha(\mathbf{r}_\perp) = \sum_{\mathbf{G}_\perp \in L_R} e^{i\mathbf{G}_\perp \mathbf{r}_\perp} \delta\mathbf{m}_\alpha(\mathbf{G}_\perp)$ and the standard relations

$$\frac{1}{V_{\mathrm{UC}}} \sum_{\mathbf{G}_\perp \in L_R} e^{i\mathbf{G}_\perp \mathbf{r}_\perp} = \delta(\mathbf{r}_\perp), \qquad \int_{V_{\mathrm{UC}}} d\mathbf{r}_\perp e^{i\mathbf{G}_\perp \mathbf{r}_\perp} = V_{\mathrm{UC}} \delta_{\mathbf{G}_\perp, 0}, \tag{21}$$

where the integral in the second equation is over the two-dimensional (primitive) unit cell of the magnetic skyrmion crystal with volume V_{UC}. The orthogonality relation can then be expressed as

$$\frac{1}{V_{\mathrm{UC}}} \int_{V_{\mathrm{UC}}} d\mathbf{r}_\perp \delta\mathbf{m}^\dagger_{\alpha'}(\mathbf{r}_\perp) i \left(\mathbf{m}_0(\mathbf{r}_\perp) \times \delta\mathbf{m}_\alpha(\mathbf{r}_\perp)\right) = \delta_{\alpha,\alpha'}. \tag{22}$$

The integrand $\mathcal{A}(\mathbf{r}_\perp) = \delta\mathbf{m}^\dagger_\alpha(\mathbf{r}_\perp) i \left(\mathbf{m}_0(\mathbf{r}_\perp) \times \delta\mathbf{m}_\alpha(\mathbf{r}_\perp)\right)$ for $\alpha = \alpha'$ has a transparent geometrical interpretation. The vector $i \left(\delta\mathbf{m}_\alpha(\mathbf{r}_\perp) \times \delta\mathbf{m}^*_\alpha(\mathbf{r}_\perp)\right) = \mathcal{A}(\mathbf{r}_\perp)\, \mathbf{m}_0(\mathbf{r}_\perp)$ (assuming that

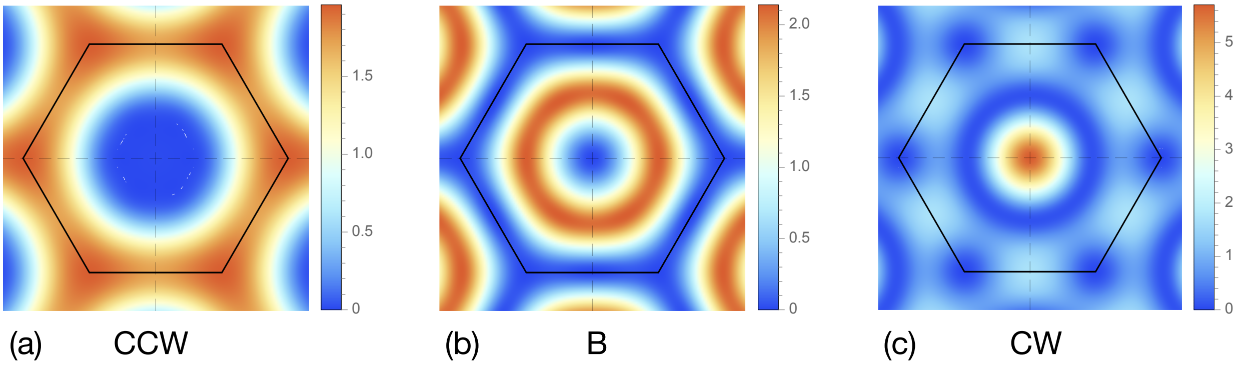

Supplementary Figure 6: **a-c**, Integrand $\mathcal{A}(\mathbf{r}_\perp) = \delta\mathbf{m}^\dagger_\alpha(\mathbf{r}_\perp) i\, (\mathbf{m}_0(\mathbf{r}_\perp) \times \delta\mathbf{m}_\alpha(\mathbf{r}_\perp))$ of the normalization condition (22) in the two-dimensional plane perpendicular to the magnetic field for the CCW, breathing and CW mode at the magnetic field $H = 0.4H_{c2}$. The black lines indicate the primitive unit cell of the magnetic skyrmion crystal.

$\mathbf{m}_0^2(\mathbf{r}_\perp) = 1$) composed of the complex spin wave eigenfunction is by definition aligned with the local magnetization $\mathbf{m}_0(\mathbf{r}_\perp)$ as both $\delta\mathbf{m}_\alpha(\mathbf{r}_\perp)$ and its complex conjugate are perpendicular to $\mathbf{m}_0(\mathbf{r}_\perp)$ in the linear spin wave approximation. In order to understand the geometrical meaning of the scalar quantity $\mathcal{A}(\mathbf{r}_\perp)$ consider the dynamical part of the magnetization attributed to the spin wave mode with quantum number α, $\delta\mathbf{m}_\alpha(\mathbf{r}_\perp, t) = \delta\mathbf{m}_\alpha(\mathbf{r}_\perp)e^{-i\omega_\alpha t} + c.c.$. The area enclosed by $\delta\mathbf{m}_\alpha(\mathbf{r}_\perp, t)$, i.e., by the local precession of the magnetization over one oscillation period is given by

$$\int_0^{2\pi/\omega_\alpha} dt\, \frac{1}{2}|\delta\mathbf{m}_\alpha(\mathbf{r}_\perp, t) \times \partial_t\delta\mathbf{m}_\alpha(\mathbf{r}_\perp, t)| = 2\pi|i\delta\mathbf{m}_\alpha(\mathbf{r}_\perp) \times \delta\mathbf{m}^*_\alpha(\mathbf{r}_\perp)| = 2\pi|\mathcal{A}(\mathbf{r}_\perp)|. \quad (23)$$

Up to a factor of 2π this can be identified with the magnitude of $\mathcal{A}(\mathbf{r}_\perp)$. Further inspections shows that $\mathcal{A}(\mathbf{r}_\perp)$ is positive and negative when the local magnetization precesses, respectively, counterclockwise and clockwise around the local equilibrium magnetization. Examples for the density distribution $\mathcal{A}(\mathbf{r}_\perp)$ can be found in Fig. 1k-m of the main text that are repeated in Supplementary Fig. 6 with a different coloring. For the important spin wave modes under consideration we find that $\mathcal{A}(\mathbf{r}_\perp)$ is always positive implying that the local precession is counterclockwise at all positions $\mathbf{r}_\perp$.

2. Analytical expression for the velocity v_∞

In order to determine the velocity v_∞ we can apply perturbation theory in the wavevector k_z. Consider first the contribution to the matrix $\mathcal{W}$ attributed to the DM interaction and stray field energy

$$\mathcal{W}_{nm}(\mathbf{K}_\perp, \mathbf{K'}_\perp; \mathbf{k}) = i\frac{\gamma}{M_s}\epsilon_{n\ell j}\mathbf{m}_{0,\ell}(\mathbf{K}_\perp - \mathbf{K'}_\perp)\left(\chi^{-1}_{\mathrm{DM},jm}(\mathbf{K'}_\perp + \mathbf{k}) + \chi^{-1}_{\mathrm{dip},jm}(\mathbf{K'}_\perp + \mathbf{k})\right) + \dots \tag{24}$$

with $\chi^{-1}_{\mathrm{DM},jm}(\mathbf{q}) = 2D\epsilon_{jnm} i\mathbf{q}_n$ and $\chi^{-1}_{\mathrm{dip},jm}(\mathbf{q}) = \mu_0 M_s^2 \frac{\mathbf{q}_j\mathbf{q}_m}{\mathbf{q}^2}$ of Supplementary Equation (2). Both contribute to first order in k_z,

$$\mathcal{W}^{(1)}_{nm}(\mathbf{K}_\perp, \mathbf{K'}_\perp; \mathbf{k}) = \tag{25}$$
$$i\frac{\gamma}{M_s}\epsilon_{n\ell j}\mathbf{m}_{0,\ell}(\mathbf{K}_\perp - \mathbf{K'}_\perp)\left(2D\epsilon_{jzm} i + \mu_0 M_s^2 \frac{\mathbf{K'}_{\perp,j}\delta_{m,z} + \mathbf{K'}_{\perp,m}\delta_{j,z}}{\mathbf{K'}^2_\perp}\Big|_{\mathbf{K'}_\perp \neq 0}\right) k_z.$$

Treating this correction in perturbation theory we obtain a correction to the spin wave frequency

$$\delta\omega_\alpha(k_z) = \langle \delta m_\alpha | \mathcal{W}^{(1)} | \delta m_\alpha \rangle = \tag{26}$$
$$= \sum_{\mathbf{G}_\perp, \mathbf{G'}_\perp, \mathbf{K'}_\perp \in L_R} \delta\mathbf{m}^\dagger_\alpha(\mathbf{G'}_\perp) i \left(\mathbf{m}_0(\mathbf{G'}_\perp - \mathbf{G}_\perp) \times (\mathcal{W}^{(1)}(\mathbf{G}_\perp, \mathbf{K'}_\perp; \mathbf{k})\delta\mathbf{m}_\alpha(\mathbf{K'}_\perp))\right).$$

This identifies the slope $v_{\alpha,\infty} = \delta\omega_\alpha / k_z$ for the mode with quantum number α.

In the following, we discuss separately the two contributions to $v_{\alpha,\infty}$ attributed to the DM interaction and the stray field energy. The expressions for $v_{\alpha,\infty}$ become particularly transparent when they are expressed as a spatial integral over the two-dimensional magnetic unit cell.

a. Non-reciprocity due to the DM interaction. The contribution due to the DM interaction can be written in the form

$$\frac{v^{\mathrm{DMI}}_{\alpha,\infty}}{v_{\mathrm{FP},\infty}} = \frac{1}{V_{\mathrm{UC}}}\int_{V_{\mathrm{UC}}} d\mathbf{r}_\perp \delta\mathbf{m}^\dagger_\alpha(\mathbf{r}_\perp) i \left(\hat{z} \times \delta\mathbf{m}_\alpha(\mathbf{r}_\perp)\right) \tag{27}$$

where $v_{\mathrm{FP},\infty} = 2\frac{g\mu_B \mu_0 H^{\mathrm{int}}_{c2}}{\hbar Q}$ is the velocity of the field-polarized phase. In the derivation we used that $\delta\mathbf{m}_\alpha(\mathbf{r}_\perp)\mathbf{m}_0(\mathbf{r}_\perp) = 0$ and $\mathbf{m}_0^2(\mathbf{r}_\perp) = 1$. Only regions in space contribute to the integral of Supplementary Equation (27) where the spin wavefunction $\delta\mathbf{m}_\alpha(\mathbf{r}_\perp)$ are in-plane, i.e., orthogonal to $\hat{z}$. Examples for the distribution of the integrand are shown in Supplementary Fig. 7.

As discussed in the main text, the integrand can be written, $\delta\mathbf{m}_\alpha^\dagger(\mathbf{r}_\perp)i\left(\hat{z}\times\delta\mathbf{m}_\alpha(\mathbf{r}_\perp)\right) = \mathcal{A}(\mathbf{r}_\perp)\mathbf{m}_{0,z}(\mathbf{r}_\perp)$, as a product of the normalized density $\mathcal{A}(\mathbf{r}_\perp)$ and $\mathbf{m}_{0,z}(\mathbf{r}_\perp)$ that varies between -1 and 1. For the important modes under consideration we find that $\mathcal{A}(\mathbf{r}_\perp) > 0$ so that it follows for the product $\mathcal{A}(\mathbf{r}_\perp)\mathbf{m}_{0,z}(\mathbf{r}_\perp) \leq \mathcal{A}(\mathbf{r}_\perp)$ at each position $\mathbf{r}_\perp$. This implies

$$\frac{v_{\alpha,\infty}^{\mathrm{DMI}}}{v_{\mathrm{FP},\infty}} \leq \frac{1}{V_{\mathrm{UC}}}\int_{V_{\mathrm{UC}}} d\mathbf{r}_\perp \mathcal{A}(\mathbf{r}_\perp) = 1 \tag{28}$$

yielding the upper bound $v_{\mathrm{FP},\infty}$ for the DM contribution $v_{\alpha,\infty}^{\mathrm{DMI}}$. We conclude that in the skyrmion crystal phase the non-reciprocity of spin waves attributed to the DM interaction cannot exceed the one of the field-polarized phase.

b. Non-reciprocity due to the stray field energy. The additional contribution attributed to the stray field energy can be expressed in the form

$$\frac{v_{\alpha,\infty}^{\mathrm{dip}}}{v_{\mathrm{FP},\infty}} = \chi_{\mathrm{con}}^{\mathrm{int}}\frac{1}{V_{\mathrm{UC}}}\int_{V_{\mathrm{UC}}} d\mathbf{r}_\perp \mathrm{Im}\{\phi_\alpha^*(\mathbf{r}_\perp)\delta\mathbf{m}_{\alpha,z}(\mathbf{r}_\perp)\} \tag{29}$$

where $\phi_\alpha(\mathbf{r}_\perp)$ is the dimensionless magnetic potential attributed to the dynamical bulk magnetic charges after Fourier transform with respect to time,

$$\phi_\alpha(\mathbf{r}_\perp) = -i\sum_{\mathbf{G}_\perp\in L_R;\mathbf{G}_\perp\neq 0} Q\frac{(\mathbf{G}_\perp\delta\mathbf{m}_\alpha(\mathbf{G}_\perp))}{\mathbf{G}_\perp^2}e^{i\mathbf{G}_\perp\mathbf{r}_\perp}. \tag{30}$$

The potential obeys the equation

$$\nabla^2\phi_\alpha(\mathbf{r}_\perp) = -Q\rho_\alpha(\mathbf{r}_\perp), \tag{31}$$

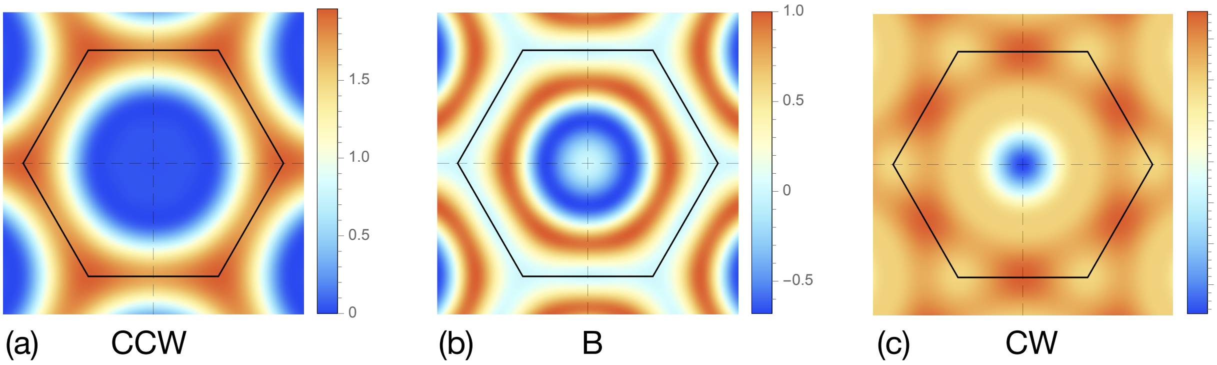

Supplementary Figure 7: **a-c**, Integrand $\delta\mathbf{m}_\alpha^\dagger(\mathbf{r}_\perp)i\left(\hat{z}\times\delta\mathbf{m}_\alpha(\mathbf{r}_\perp)\right)$ of $v_{\alpha,\infty}^{\mathrm{DMI}}$, see Supplementary Equation (27), in the two-dimensional plane perpendicular to the magnetic field for the CCW, breathing and CW mode at the magnetic field $H = 0.4H_{c2}$.

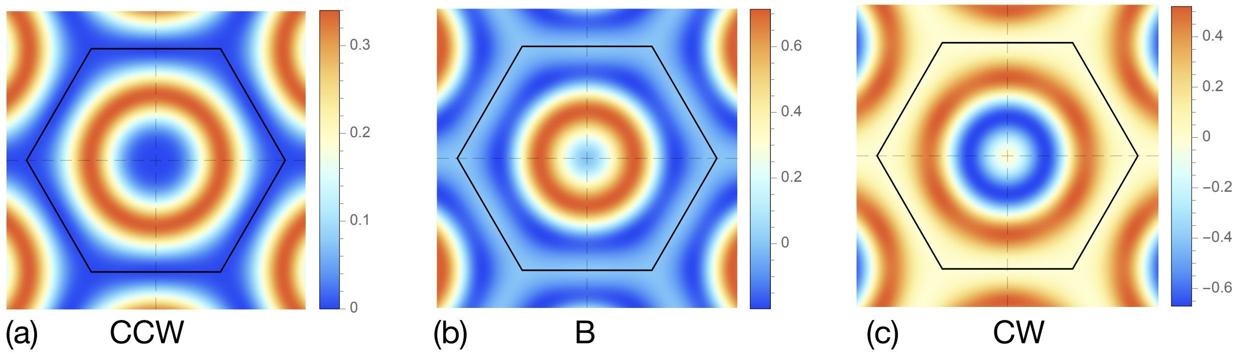

Supplementary Figure 8: **a-c**, Integrand $\text{Im}\{\phi^*_\alpha(\mathbf{r}_\perp)\delta\mathbf{m}_{\alpha,z}(\mathbf{r}_\perp)\}$ of $v^{\text{dip}}_{\alpha,\infty}$, see Supplementary Equation (29), in the two-dimensional plane perpendicular to the magnetic field for the CCW, breathing and CW mode at $H = 0.4H_{c2}$.

with the dynamical magnetic charge density $\rho_\alpha(\mathbf{r}_\perp) = -\nabla\delta\mathbf{m}_\alpha(\mathbf{r}_\perp)$ in the bulk of the sample.

Even in the limit $\mathbf{k}_\perp = 0$ and $k_z \to 0$, the spin wave excitation of the skyrmion crystal generates dynamically a spatially modulated magnetic potential, $\phi_\alpha(\mathbf{r}_\perp)$, that periodically depends on the in-plane position $\mathbf{r}_\perp$. This potential generates a dynamic dipolar field. In the limit $k_z \to 0$, this field lies in the plane, $\mathbf{H}_{\text{dip},\alpha}(\mathbf{r}_\perp) = -(M_s/Q)\nabla\phi_\alpha(\mathbf{r}_\perp)$, as the potential does not depend on the spatial z-coordinate. However, at a small but finite k_z there exists a finite spatially modulated z-component of the stray field whose Fourier transform to linear order in k_z is given by $-\frac{M_s}{Q} i k_z \phi_\alpha(\mathbf{r}_\perp)$. This leads to a stray field energy density proportional to $k_z(-i\phi_\alpha(\mathbf{r}_\perp)\delta\mathbf{m}^*_{\alpha,z}(\mathbf{r}_\perp) + \text{c.c.})$ that eventually accounts for the integrand of Supplementary Equation (29).

Examples for the $\mathbf{r}_\perp$-dependence of this integrand are shown in Supplementary Fig. 8. It is only finite in regions where the spin wave function possesses a finite z-component, i.e., the local magnetization oscillates out-of-plane because it derives from a coupling to the z-component of the dipolar stray field. Consequently, the integrand vanishes at the center and the edges of the unit cell where the equilibrium magnetization is aligned with the z axis.

The importance of dynamic dipolar interactions for the nonreciprocity of spin excitations has been pointed out before for other non-collinear spin textures, see Supplementary Refs. [17–19].

	$\frac{v_{\alpha,\infty}^{\rm DMI}}{v_{\rm FP,\infty}}$	$\frac{v_{\alpha,\infty}^{\rm dip}}{v_{\rm FP,\infty}}$	$\frac{v_{\alpha,\infty}^{\rm DMI}+v_{\alpha,\infty}^{\rm dip}}{v_{\rm FP,\infty}}$
CCW	0.83	0.25	1.08
B	0.24	0.21	0.45
CW	0.07	0.10	0.17

Supplementary Table I: Non-reciprocal velocities of the bulk spin wave dispersion for the CCW, B, and CW mode for the magnetic field $H = 0.4 H_{\rm c2}$, that is attributed to the DM interaction, $v_{\alpha,\infty}^{\rm DMI}$, and to the stray field energy, $v_{\alpha,\infty}^{\rm dip}$, see Supplementary Equations (27) and (29).

3. Numerical values for the velocities $v_{\alpha,\infty}^{\rm DMI}$ and $v_{\alpha,\infty}^{\rm dip}$

The numerically evaluated values for the velocities attributed to the DM interaction, $v_{\alpha,\infty}^{\rm DMI}$, and to the stray field energy, $v_{\alpha,\infty}^{\rm dip}$, at the magnetic field $H = 0.4 H_{\rm c2}$ are listed in Supplementary Table I. The sum of the two velocities listed in the last column is consistent with the numerically evaluated spectrum shown in Supplementary Fig. 5. Note that this value even exceeds $v_{\rm FP,\infty}$ for the CCW mode, which is only possible due to the contribution of the stray field energy as $v_{\alpha,\infty}^{\rm DMI} \leq v_{\rm FP,\infty}$. The dipolar contribution $v_{\infty}^{\rm dip}$ for the three modes is substantial and corresponds to 23%, 47%, and 59% for the CCW, breathing and CW mode, respectively. The strength of $v_{\alpha,\infty}^{\rm dip}$ is weighted by the parameter $\chi_{\rm con}^{\rm int}$, see Supplementary Equation (29), that is larger for Cu_2OSeO_3, $\chi_{\rm con}^{\rm int} = 1.76$, than for MnSi, $\chi_{\rm con}^{\rm int} = 0.34$ [3]. Consequently, the dipolar contribution is particularly important for the material Cu_2OSeO_3 as $\chi_{\rm con}^{\rm int}$ is relatively large.

---

[1] S. Seki, X. Z. Yu, S. Ishiwata, Y. Tokura. Observation of Skyrmions in a Multiferroic Material. Science **336**, 198 (2012).

[2] T. Adams, A. Chacon, M. Wagner, A. Bauer, G. Brandl, B. Pedersen, H. Berger, P. Lemmens, C. Pfleiderer. Long-Wavelength Helimagnetic Order and Skyrmion Lattice Phase in Cu_2OSeO_3. Phys. Rev. Lett. **108**, 237204 (2012).

[3] T. Schwarze, J. Waizner, M. Garst, A. Bauer, I. Stasinopoulos, H. Berger, C. Pfleiderer, D. Grundler. Universal helimagnon and skyrmion excitations in metallic, semiconducting and insulating chiral magnets. Nature Mater. **14**, 478 (2015).

[4] V. Vlaminck, M. Bailleul. Spin-wave transduction at the submicrometer scale: Experiment and modeling. Phys. Rev. B **81**, 014425 (2010).

[5] V. Vlaminck, M. Bailleul. Current-Induced Spin-Wave Doppler Shift. Science **322**, 410 (2008).

[6] C. Liu *et al.* Long-distance propagation of short-wavelength spin waves. Nat. Comm. **9**, 738 (2018).

[7] S. Maendl, L. Stasinopoulos, D. Grundler. Spin waves with large decay length and few 100?nm wavelengths in thin yttrium iron garnet grown at the wafer scale. Appl. Phys. Lett. **111**, 012403 (2017).

[8] L. J. Cornelissen, J. Liu, R. A. Duine, J. Ben Youssef, B. J. van Wees. Long-distance transport of magnon spin information in a magnetic insulator at room temperature. Nature Phys. **11**, 1022 (2015).

[9] H. Yu *et al.* Magnetic thin-film insulator with ultra-low spin wave damping for coherent nanomagnonics. Sci. Rep. **4**, 6848 (2014).

[10] J. Waizner. Spin wave excitations in magnetic helices and skyrmion lattices. (PhD thesis, Universität zu Köln (2016), http://kups.ub.uni-koeln.de/7937).

[11] M. Garst, J. Waizner, and D. Grundler. Collective spin excitations of helices and magnetic skyrmions: review and perspectives of magnonics in non-centrosymmetric magnets. J. Phys. D: Appl. Phys. **50**, 293002 (2017).

[12] M. Kugler, G. Brandl, J. Waizner, M. Janoschek, R. Georgii, A. Bauer, K. Seemann, A. Rosch, C. Pfleiderer, P. Böni, and M. Garst. Band Structure of Helimagnons in MnSi Resolved by Inelastic Neutron Scattering. Phys. Rev. Lett. **115**, 097203 (2015).

[13] T. Weber, J. Waizner, G. Tucker, R. Georgii, M. Kugler, A. Bauer, C. Pfleiderer, M. Garst, and P. Böni. Field dependence of nonreciprocal magnons in chiral MnSi. Phys. Rev. B **97**, 224403 (2018).

[14] X. Xing, Y. Zhou, H. B. Braun. Skyrmion Tubes as Magnonic Waveguides. Preprint at https://arxiv.org/abs/1901.00253 (2019).

[15] S.-Z. Lin, J.-X. Zhu, and A. Saxena. Kelvin modes of a skyrmion line in chiral magnets and the associated magnon transport. Phys. Rev. B **99**, 140408(R) (2019).

[16] V. P. Kravchuk, U. K. Rößler, J. van der Brink, M. Garst. Solitary wave excitations of skyrmion strings in chiral magnets. Preprint at https://arxiv.org/abs/1902.01420 (2019).

[17] R. Camley. Nonreciprocal surface waves. Surf. Sci. Rep. **7**, 103 (1987).

[18] J. A. Otálora *et al.* Curvature-Induced Asymmetric Spin-Wave Dispersion. Phys. Rev. Lett. **117**, 227203 (2016).

[19] Y. Henry, D. Stoeffler, J.-V. Kim, and M. Bailleul. Unidirectional spin-wave channeling along magnetic domain walls of Bloch type. Phys. Rev. B **100**, 024416 (2019).
